# LINC01578 affects the radiation resistance of lung cancer cells through regulating microRNA-216b-5p/TBL1XR1 axis

**DOI:** 10.1080/21655979.2022.2051881

**Published:** 2022-04-27

**Authors:** Peirong Wang, Linchun Ke, Chuanshu Cai, Feng Dong

**Affiliations:** Department of Radiotherapy, Cancer Center, The First Affiliated Hospital of Fujian Medical University, Fuzhou, China

**Keywords:** Non-small cell lung cancer, LINC01578, Transducin (beta)-like 1 X-linked receptor 1, biological function, radiation resistance

## Abstract

Radiation resistance largely limits the survival of patients with non-small-cell lung cancer (NSCLC). To understand the mechanism underlying radiation resistance, we explored the influence of LINC01578 in radiation-resistant NSCLC cells. LINC01578, miR-216b-5p and Transducin (beta)-like 1 X-linked receptor 1 (TBL1XR1) expression was evaluated in patients with NSCLC, and their correlation with patients’ prognosis was examined. Radiation-resistant NSCLC cell line (A549-RR) was induced and treated with oligonucleotide or plasmid transfection, and cell biological functions were captured. The interplay between LINC01578, miR-216b-5p and TBL1XR1 was clarified. NSCLC patients showed high LINC01578 and TBL1XR1 expression, and low miR-216b-5p expression, which was correlated to shorter patients’ prognosis, respectively. LINC01578 or TBL1XR1 deficiency or miR-216b-5p elevation suppressed the functional activities of A549-RR cells. LINC01578 suppression elevated miR-216b-5p expression, consequently leading to the down-regulation of TBL1XR1. miR-216b-5p silencing or TBL1XR1 overexpression compromised LINC01578 knockdown’s effects on radiation resistance of A549-RR cells. In brief, LINC01578 suppresses miR-216b-5p and enhances TBL1XR1 expression, thus to promote biological functions of radiation-resistant NSCLC cells.

## Graphical abstract

**Figure:**
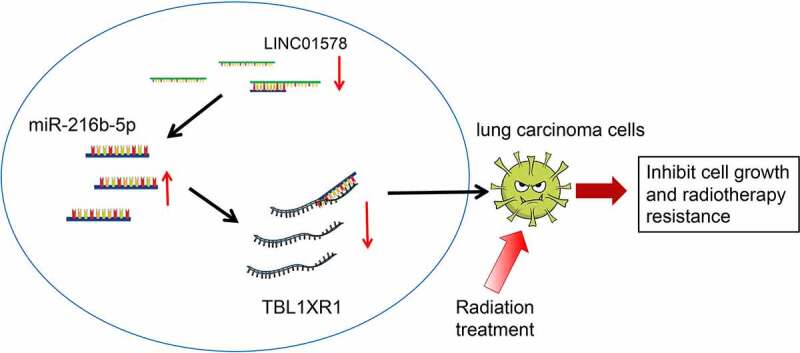

## Highlights

- NSCLC patients show high LINC01578 expression.
- LINC01578 deficiency suppresses the functional activities of A549-RR cells.
- LINC01578 suppression elevates miR-216b-5p expression.
- miR-216b-5p elevation re-sensitizes A549-RR cells to radiation.
- miR-216b-5p targets TBL1XR1.

## Introduction

In the global scope, the number of lung cancer cases and deaths is rising. In 2020, the age-standardized rate in China is 34.8%, and 815563 cases were diagnosed as lung cancer. NSCLC is firstly diagnosed by histological classification based on morphological evaluation of tumors, and then the auxiliary immunohistochemistry method [1]. The growing understanding of the molecular basis that drives lung cancer has enabled the development of therapies targeting specific genomic alterations. Immunotherapy, mainly through the use of checkpoint inhibitors, has shown promising results in the treatment of lung cancer [2]. Radiotherapy will remain nonselective and indiscriminate to eradicate persistent and drug-resistant tumor stem cells [3]. In tumors resistant to chemotherapy and radiotherapy, successful treatment for lung cancer is still challenging [4].

Long noncoding RNA (lncRNA) is a non-protein coding RNA that participates in a wide range of biological processes of cancer [^5–7^]. LINC01578 is an evolutionarily conserved RNA, which can co-transcribe and regulate target genes by guiding CHD2 helicase to interact with new transcripts. LINC01578 presents a potential prognostic biomarker and a therapeutic target for metastatic colon cancer [8]. Nevertheless, not much was known about LINC01578ʹs role in NSCLC.

MicroRNA (miR) is a highly conserved RNA that maintain cell homeostasis. A balanced physiological environment requires proper control of miRNA expression, because these small molecules affect almost all genetic pathways with a wide range of target genes [9]. By acting as a tumor suppressor or an oncogene, the dysregulation of miR expression is associated with various cancers [10,11]. miR-216b-5p generally confers inhibitive effects on cancer development [12,13], and it has been reported that miR-216b-5p could interact with certain lncRNA, and even could compromise the oncogenic activities of lncRNAs, such as LINC00152 and LINC00518 [14,15].

Transducin (beta)-like 1 X-linked receptor 1 (TBL1XR1) is an evolutionarily conserved protein. TBL1XR1 is up-regulated in a variety of solid tumors, including lung squamous cell carcinoma and is related to tumor stage and metastasis [16]. TBL1XR1 suggests a prognostic value and also an adjuvant therapeutic target for chemoresistance in the treatment of NSCLC [17]. For lung squamous cell carcinoma, TBL1XR1 up-regulation generates a mesenchymal phenotype while TBL1XR1 down-regulation produces an epithelial phenotype [18].

We in advance predicted the potential interplay between LINC01578, miR-216b-5p and TBL1XR1 on the bioinformatics website. Based on that, we investigated radiation resistance in NSCLC, mainly focusing on the influence of LINC01578/miR-216b-5p/TBL1XR1 axis on biological functions of radiation-resistant cancer cells. It was inferred that LINC01578 induced radiation resistance of NSCLC cells through the network of miR-216b-5p/TBL1XR1.

## Materials and methods

### Ethical approval

All patients gave informed consent, and this study was approved by the ethics committee of The First Affiliated Hospital of Fujian Medical University.

### Experimental tissue sampling

One hundred and twenty pairs of lung cancer tissue and adjacent normal tissue were collected from NSCLC patients in The First Affiliated Hospital of Fujian Medical University. There were 67 males and 53 females, aged 40–67 years old. The survival time of all patients was calculated from the time of diagnosis, and the 5-year follow-up ended in April 2020.

### Cell culture

P12K Medium (Sigma, Shanghai, China) was employed to cultivate human lung cancer cell line, A549. The medium contained 10% fetal bovine serum (FBS), 100 U/mL penicillin sodium and 100 mg/mL streptomycin [19].

### Cell transfection

sh-LINC01578, miR-216b-5p-mimic, si-TBL1XR1 and their negative controls (sh-NC, mimic-NC, si-NC) were all synthesized by GenePharma (Shanghai, China) and transfected into radiation-resistant cells, respectively. Four combined transfection plans were designed, including sh-LINC01578 + miR-216b-5p-inhibitor, sh-LINC01578 + inhibitor-NC, sh-LINC01578 + oe-TBL1XR1 and sh-LINC01578 + oe-NC. Among which, miR-216b-5p inhibitor, oe-TBL1XR1and their negative controls (inhibitor-NC, oe-NC) were all synthesized by GenePharma. Transfection was performed through Lipofectamine 3000 (Invitrogen, Shanghai, China) [20].

### Establishment of radiation-resistant cell line

To induce radiation resistance, Gammacell®40 Exactor (AECL, Ontario, Canada) was used, and 137Cs gamma rays were irradiated at 1 Gy/min. Parental A549 cells were exposed to 0–10 Gy in the observation test, and 5 Gy was selected for subsequent tests. A549 cells were sequentially exposed to increasing doses of radiation to produce a radiation-resistant NSCLC cell line (A549-RR). The parental radiation-sensitive cell line was defined as A549-RS [21].

### Proliferation assay

According to the protocol of Cell Counting Kit-8 (Dojindo, Japan), cells were seeded in 96-well plates and subjected to analysis of absorbance using an enzyme immunoassay analyzer (Bio-rad, CA, USA) [22].

### Colony formation assay

  Cells were seeded into 6-well plates at a density of 1 x 10^3^ cells/well. During the 14-d incubation, the medium was renewed every 3 d, and colonies were formed. Lastly, 0.1% crystal violet staining was performed and the colonies were manually counted [23].

### Wound healing assay

Cells on 12-well plates at 2 × 10^5^ cells/well were wounded by a sterile 200 μL pipette tip. Cells were washed with PBS to remove non-adherent cells. Cells were then incubated with serum-free F12K medium for 24 h at 37°C. At 0 and 24 h, cell images were collected and wound healing rate was examined using ImageJ software [24].

### Invasion assay

Cells were cultured in Boyden Chamber 24-well plates (Corning Costar, NY, USA) at 5 × 10^4^ cells/well. The upper chamber was coated with Matrigel (BD, Biosciences, NJ, USA) and contained serum-free medium, and the lower one contained 20% FBS-F12K medim. After 48 h, 0.1% crystal violet staining was used to mark the invasive cells which were subsequently captured by an optical microscope (Nikon, Japan) and counted by Image Pro Plus 6.0 [25].

### Apoptosis analysis

Cells were stained with Annexin-V-fluorescein isothiocyanate Apoptosis Detection Kit (BD Bioscience). Data acquisition was carried out by FACSCalibur flow cytometer (BD Biosciences) and apoptosis rate analysis was by CellQuest 3.0 software (BD Biosciences) [26].

### Quantitative PCR

Total RNA was isolated with Trizol reagent (Invitrogen) and used for reverse transcription via PrimeScript RT Master Mix and Mir‐X miRNA First‐Strand Synthesis Kit (TaKaRa, Dalian, China) . LINC01578, miR-216b-5p and TBL1XR1 expression was examined using SYBR Premix Ex Taq II Kit (Takara), with U6 as the internal reference for miR-216b-5p, and glyceraldehyde-3-phosphatedehydrogenase (GAPDH) as that for LINC01578 and TBL1XR1. Supplementary Table S1 lists the primer sequences for PCR. The 2^−ΔΔCt^ method was adopted for expression calculation.

### Immunoblotting

Tissue and cells were lysed using radio-immunoprecipitation assay lysis buffer (Sangon, Shanghai, China) to collect proteins. After sodium dodecyl sulfate polyacrylamide gel electrophoresis, proteins were loaded on a polyvinylidene fluoride membrane (Bio-Rad, CA, USA) and blocked with 5% skim milk for incubating with primary antibodies TBL1XR1 (1:5000) and GAPDH (1:500; Abcam), and with horseradish peroxidase-labeled secondary antibody (1:2000; Abcam). Band visualization was performed by ECLPlus Western Blot. Antibodies were supplied by Abcam (MA, USA) [27].

### RNA immunoprecipitation (RIP)

RIP kit (Millipore, MA, USA) was used for RIP assay. Cell lysate was combined with magnetic beads conjugated with Ago2 antibody (1:30; Abcam) or IgG antibody (Abcam). After incubation with proteinase K, the immunoprecipitated RNA was utilized to measure LINC01578, miR-216b-5p and TBL1XR1 levels through quantitative PCR [28].

### Luciferase reporter assay

LINC01578-wild-type (WT), LINC01578-mutant (MUT), TBL1XR1-WT and TBL1XR1-MUT 3'-untranslated region (UTR) sequences were cloned into pmirGLO plasmids (Promega Corporation). A549-RR cellswere transfected with the reporter and miR-216b-5p mimic or mimic NC. Cell luciferase activity was analyzed in dual luciferase reporter system (Promega, WI, USA) [29].

### Statistical evaluation

Data acquisition was conducted with SPSS 21.0 (IBM, NY, USA) and GraphPad Prism 6 software (GraphPadSoftware, San Diego, CA, USA). Data normal distribution was verified by Kolmogorov-Smirnov test, and the values were reported as mean ± standard deviation. *t* -test was suitable for comparative analysis of two-group data, analysis of variance for that of multi-group data and Tukey’s post hoc test for that of pairwise data. Enumeration data, presented by rate or percentage, were analyzed by chi-square test. Correlation analyses were performed using Pearson statistics. *P* indicated two-sided test, and its value less than 0.05 meant statistically significance.

## Results

### Prognostic role of LINC01578 in NSCLC

**Figure 1.**
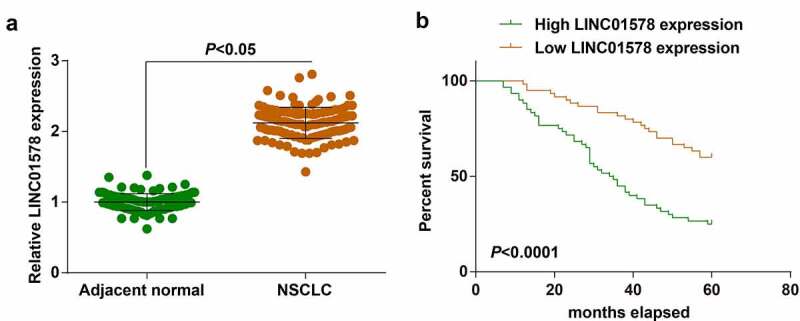
Prognostic role of LINC01578 in LC. (a). LINC01578 expression in NSCLC tissues; (b). Effect of LINC01578 expression on prognosis of patients. Measurement data were presented as mean ± standard deviation.

In 120 patients with NSCLC, examination of LINC01578 expression was conducted: higher LINC01578 was found in NSCLC tissues than in adjacent normal tissues (Figure 1(a)). Clinical correlation between LINC01578 expression and patients’ parameters was studied. We divided all patients into high expression group (n = 60) and low expression group (n = 60) based on the median LINC01578 expression. Using chi-square test, it was proved that LINC01578 high expression was correlated with tumor size, lymph node metastasis and TNM stage (Supplementary Table S2). We further studied the impact of LINC01578 on patients’ prognosis using Kaplan-Meier analysis: high LINC01578 expression indicated a decreased survival rate of patients (Figure 1(b)).

### A549-RR cells show enhanced radiation resistance

**Figure 2.**
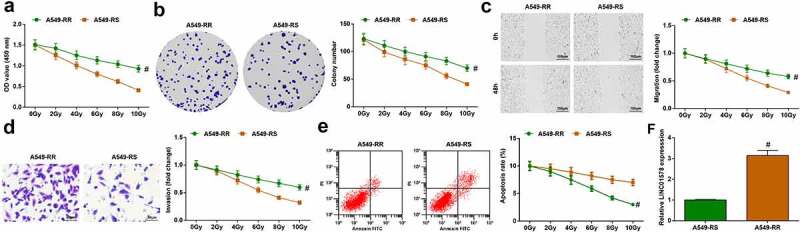
A549-RR cells have stronger resistance to radiation. (a-b). A549-RR cells had stronger proliferation than A549-RS cells; (c). A549-RR cells had stronger migration ability than A549-RS cells; (d). A549-RR cells had stronger invasion ability than A549-RS cells; (e). A549-RR cells had less apoptosis than A549-RS cells; (f). LINC01578 expression in A549-RS and A549-RR cells; Measurement data were presented as mean ± standard deviation. # *vs*. A549-RS cells, *P* < 0.05.

A549-RR and A549-RS cells were exposed to irradiation at 0–10 Gy. Biological function tests found that as the radiation dose increased, proliferation, colony formation ability, migration and invasion of A549-RR and A549-RS cells decreased; irradiated at the same dose, A549-RR cells had stronger cell proliferation, migration and invasion ability than A549-RS cells (Figure 2(a-d)). Under different radiation doses, with the increase of radiation dose, the apoptosis rate of A549-RR and A549-RS cells showed an elevated trend; A549-RR cells had a lower apoptosis rate than A549-RS cells when irradiated at the same dose (Figure 2(e)). LINC01578 expression was detected in the two cell lines, exhibiting a higher level in A549-RR cells than A549-RS cells (Figure 2(f)). All in all, radiation-resistant NSCLC cells have stronger resistance to radiation than parental radiation-sensitive cells.

### Depleting LINC01578 reduces radiation resistance of A549-RR cells

**Figure 3.**
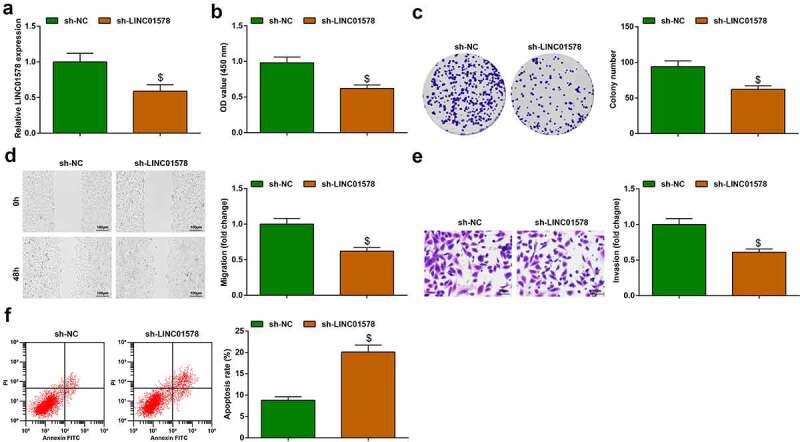
Depleting LINC01578 reduces radiation resistance of A549-RR cells. (a). sh-LINC01578 transfection reduced LINC01578 expression in A549-RR cells; (b-c). sh-LINC01578 reduced A549-RR cell proliferation; (d). sh-LINC01578 reduced A549-RR cell migration ability; (e). sh-LINC01578 reduced A549-RR cell invasion ability; (f). sh-LINC01578 elevated A549-RR cell apoptosis; Measurement data were presented as mean ± standard deviation. $ *vs*. the sh-NC group, *P* < 0.05.

A549-RR cells were successfully transfected with sh-LINC01578, as reflected by LINC01578 expression reduction tested by quantitative PCR (Figure 3(a)). As the results of LINC01578 expression reduction, impairments of proliferation, invasion and migration and augment of apoptosis rate were seen in A549-RR cells (Figure 3(b-f)). To briefly conclude, LINC01578 deficiency reduces the radiation resistance of radiation-resistant NSCLC cells.

### Elevating miR-216b-5p expression re-sensitizes A549-RR cells to radiation

**Figure 4.**
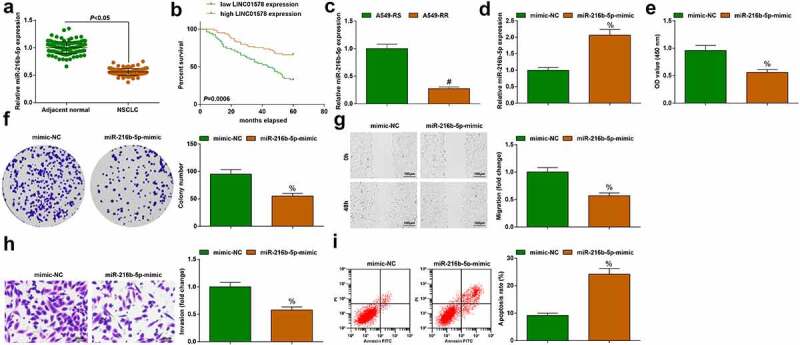
Elevating miR-216b-5p re-sensitizes A549-RR cells to radiation. (a). miR-216b-5p expression in NSCLC tissues; (b). Kaplan-Meier analysis of survival rate; (c). miR-216b-5p expression in A549-RS and A549-RR cells; (d). miR-216b-5p-mimic transfection elevated miR-216b-5p expression in A549-RR cells; (e-f). miR-216b-5p-mimic reduced A549-RR cell proliferation; (g). miR-216b-5p-mimic reduced A549-RR cell migration ability; (h). miR-216b-5p-mimic reduced A549-RR cell invasion ability; (i). miR-216b-5p-mimic elevated A549-RR cell apoptosis; Measurement data were presented as mean ± standard deviation. % *vs*. the mimic-NC group, *P* < 0.05.

In the cancer tissue and adjacent normal tissue of NSCLC patients, miR-216b-5p expression was compared by quantitative PCR: miR-216b-5p down-regulation was detected in the cancer tissue versus adjacent normal tissue (Figure 4(a)).

The results obtained by Kaplan-Meier analysis showed that the survival rate of patients with high expression of miR-216b-5p was increased (Figure 4(b)). Moreover, miR-216b-5p expression was determined to be lower in A549-RR cells than A549-RS cells (Figure 4(c)). For A549-RR cells, transfection with miR-216b-5p-mimic was implemented, with mimic NC as the control: miR-216b-5p-mimic successfully elevated cellular miR-216b-5p expression (Figure 4(d)). In miR-216b-5p-overexpressed A549-RR cells, it was clearly examined that proliferation, migration and invasion abilities were destructed and apoptosis rate was promoted (Figure 4(e-i)). Overall, miR-216b-5p up-regulation re-sensitizes radiation-resistant NSCLC cells to radiation.

### Binding regulation between LINC01578 and miR-216b-5p

**Figure 5.**
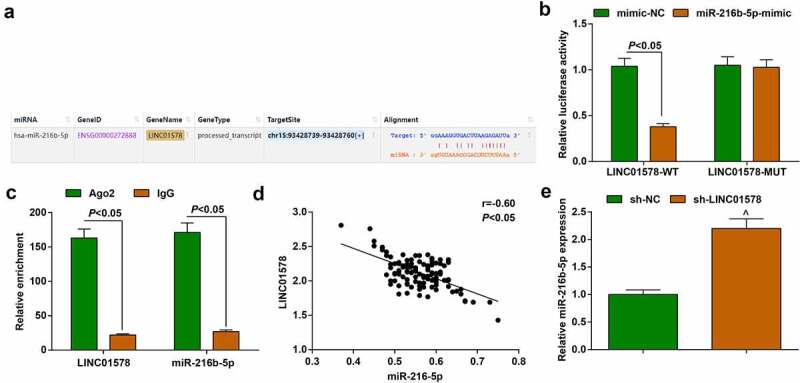
Binding regulation between LINC01578 and miR-216b-5p. (a). Wild‐type (WT) and mutated‐type (MUT) sequences of the putative binding sites between LINC01578 and miR-216b-5p; (b-c). The combination of LINC01578 and miR-216b-5p analyzed by luciferase assay and RIP experiment; (d). Correlation between LINC01578 and miR-216b-5p levels; (e). sh-LINC01578 reduced miR-216b-5p expression in A549-RR cells. Measurement data were presented as mean ± standard deviation. $ *vs*. the sh-NC group, *P* < 0.05.

To specify the molecular interplay between LINC01578 and miR-216b-5p, we employed starbase website and observed the binding site between LINC01578 and miR-216b-5p     (Figure 5(a)). To further understand their relation, we co-transfected LINC01578-WT or LINC01578-MUT vectors with miR-216b-5p-mimic or mimic-NC into A549-RR cell line, and tested that the combination of LINC01578-WT and miR-216b-5p-mimic decreased the luciferase activity (Figure 5(b)). Also, we conducted RIP experiment and experimentally observed that the enrichment levels of LINC01578 and miR-216b-5p were synchronously augmented by anti-Ago2 antibody (Figure 5(c)).

Pearson analysis tested that LINC01578 and miR-216b-5p were negatively correlated in patients’ cancer tissue samples (Figure 5(d)). miR-216b-5p expression in cells stably transfected with sh-LINC01578 was analyzed by quantitative PCR, and the results reflected that miR-216b-5p expression was elevated, further proving the regulatory relationship between LINC01578 and miR-216b-5p (Figure 5(e)).

### TBL1XR1 deficiency blocks the development of A549-RR cells

**Figure 6.**
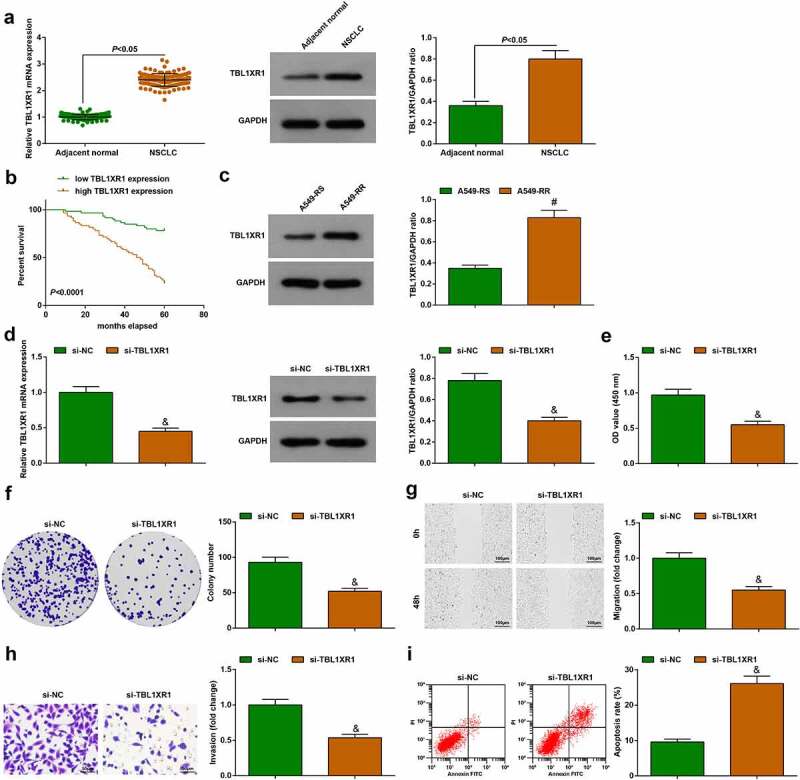
TBL1XR1 deficiency blocks the development of A549-RR cells. (a). TBL1XR1 mRNA and protein expression in 120 pairs of tissue; (b). Kaplan-Meier analysis of survival rate; (c). TBL1XR1 protein expression in A549-RS and A549-RR cells; (d). si-TBL1XR1 transfection suppressed TBL1XR1 mRNA and protein expression in A549-RR cells; (e-f). si-TBL1XR1 reduced A549-RR cell proliferation; (g). si-TBL1XR1 reduced A549-RR cell migration ability; (h). si-TBL1XR1 reduced A549-RR cell invasion ability; (i). si-TBL1XR1 elevated A549-RR cell apoptosis; Measurement data were presented as mean ± standard deviation. & *vs*. the si-NC group, *P* < 0.05.

Quantitative PCR and immunoblotting showed that TBL1XR1 was up-regulated in cancer tissue samples of patients (Figure 6(a)). Kaplan-Meier analysis showed that patients with high-expressing TBL1XR1 had reduced survival rate (Figure 6(b)). TBL1XR1 protein expression presented an increase in A549-RR cells compared to A549-RS cells (Figure 6(c)). In A549-RR cells, transfection with si-TBL1XR1 reduced TBL1XR1 expression (Figure 6(d)). From the same experiments, it could be seen that proliferation, migration, and invasion capabilities decreased, the apoptosis rate increased in TBL1XR1-depleted A549-RR cells (Figure 6(e-i)). Thus, TBL1XR1 deficiency blocks the malignant behaviors of NSCLC radiation-resistant cells.

### Targeting regulation between miR-216b-5p and TBL1XR1

**Figure 7.**
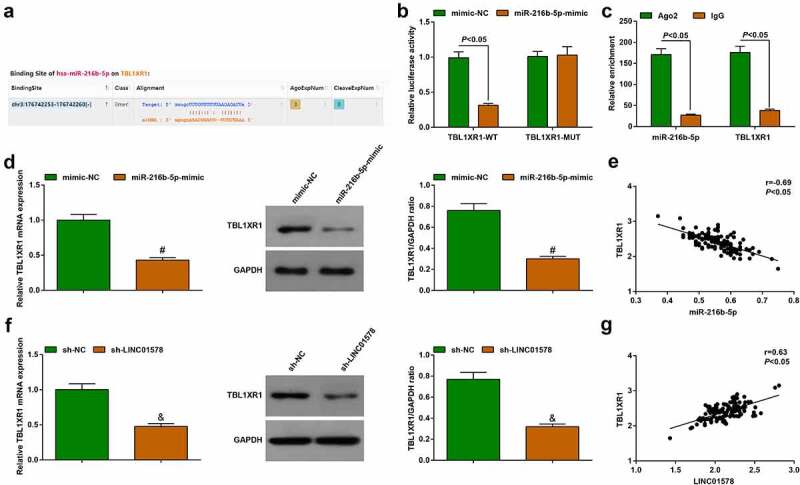
Targeting regulation between miR-216b-5p and TBL1XR1. (a). TBL1XR1 wild‐type (WT) and mutated‐type (MUT) sequences of the putative binding sites between GRAMD1 and miR-216b-5p; (b-c). The combination of miR-216b-5p and TBL1XR1 analyzed by luciferase assay and RIP experiment; (d). miR-216b-5p mimic decreased TBL1XR expression; E. Correlation between miR-216b-5p and TBL1XR1 levels; (f). sh-LINC01578 reduced TBL1XR expression; (g). Correlation between LINC01578 and TBL1XR1 levels. Measurement data were presented as mean ± standard deviation. % *vs*. the mimic-NC group, *P* < 0.05; $ *vs*. the sh-NC group, *P* < 0.05.

To verify whether there was a targeting relationship between miR-216b-5p and TBL1XR1, we first tested the possible targeting relationship between the two through the starBase website, confirming a binding region between miR-216b-5p and TBL1XR1     (Figure 7(a)). Similarly, we performed luciferase reporter experiment and RIP experiment to further verify the targeting interplay between the two: miR-216b-5p mimic inhibited the luciferase activity of TBL1XR1-WT, but had no significant effect on that of TBL1XR1-MUT (Figure 7(b)); moreover, the enrichment levels of miR-216b-5p and TBL1XR1 mRNA were simultaneously elevated by antiAgo2(Figure 7(c)).

Changes of TBL1XR1 expression were seen in stably transfected A549-RR cells: miR-216b-5p-mimic or sh-LINC01578 suppressed TBL1XR1 expression (Figure 7(d,f)). Gene expression correlation analysis in patients’ cancer tissue samples showed that miR-216b-5p was negatively correlated with TBL1XR1 (Figure 7(e)) and LINC01578 was positively correlated with TBL1XR1 (Figure 7(g)).

### LINC01578 mediates TBL1XR1 expression through miR-216b-5p and affects LC radiation resistance

**Figure 8.**
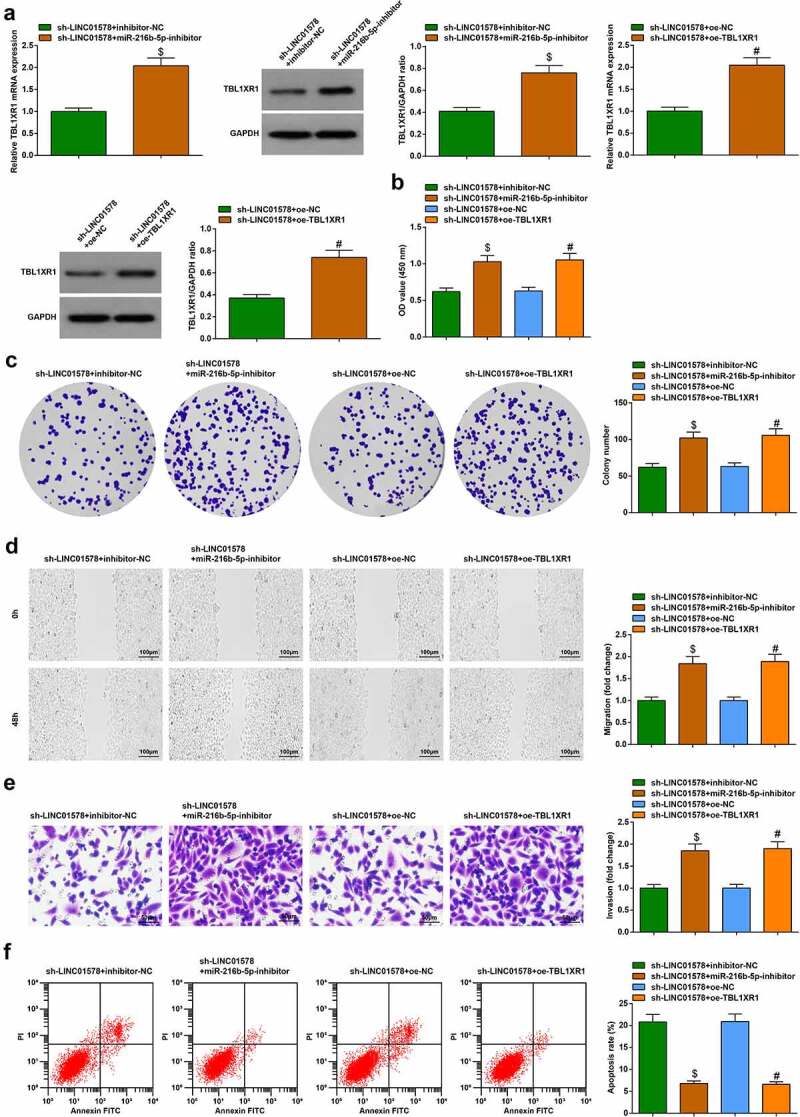
LINC01578 mediates TBL1XR1 expression through miR-216b-5p and affects LC radiation resistance. (a-b). miR-216b-5p inhibitor or oe-TBL1XR1 elevated TBL1XR1 expression in sh-LINC01578-modified A549-RR cells; (c-d). miR-216b-5p inhibitor or oe-TBL1XR1 augmented sh-LINC01578-modified A549-RR cell proliferation; (e). miR-216b-5p inhibitor or oe-TBL1XR1 augmented sh-LINC01578-modified A549-RR cell migration ability; (f). miR-216b-5p inhibitor or oe-TBL1XR1 augmented sh-LINC01578-modified A549-RR cell invasion ability; (g). miR-216b-5p inhibitor or oe-TBL1XR1 suppressed sh-LINC01578-modified A549-RR cell apoptosis; Measurement data were presented as mean ± standard deviation. $ *vs*. the sh-LINC01578 + inhibitor-NC group, *P* < 0.05; # *vs*. the sh-LINC01578 + oe-NC group, *P* < 0.05.

Four combined transfection designs were executed to verify that LINC01578 modifies TBL1XR1 by regulating miR-216b-5p in NSCLC. The transfection efficiency of different designs was first verified (Figure 8(a-b)). Alternation of cell biology was noticed: LINC01578 low expression-induced growth suppression of A549-RR cells was restrained by miR-216b-5p inhibition or TBL1XR1 overexpression (Figure 8(c-g)). Evidently, LINC01578 mediates TBL1XR1 expression through miR-216b-5p and affects the biological functions of radiation-resistant NSCLC cells.

## Discussion

Although modern photon radiation technology is widely used, as many as 25% of patients have experienced local recurrence [30]. To make matters worse, the intrinsic and acquired radiation resistance is a thorny issue in the treatment of NSCLC. In the present research, efforts were made to overcome radiation resistance in NSCLC, ultimately verifying that LINC01578 suppression impaired the malignant phenotype of radiation-resistant NSCLC cells via up-regulating miR-216b-5p and down-regulating TBL1XR1.

Loss of LINC01578 increases Chd2 mRNA and protein levels, which in turn leads to transcriptional interference by inhibiting the promoter of downstream highly expressed genes [31]. LINC01578 has been examined to be overexpressed in colon cancer patients and is correlated with patients’ poor prognosis, and LINC01578 is an inducer for cell proliferation [8]. Accordant with the previous study, we checked the up-regulation of LINC01578 in patients with NSCLC, and the correlation between LINC01578 expression and patients’ tumor node metastasis staging, lymph node metastasis and prognosis. We further concluded from function assays that silenced LINC01578 alone repressed the pro-tumor activities of radiation-resistant NSCLC cells. Compared with the A549-RS cells, LINC01578 expression in A549-RR cells was increased, so we speculate that overexpressed LINC01578 can increase the radiation resistance of cells.

It is reported that miR-216b-5p is lowly expressed in patients with serous epithelial ovarian cancer, indicating a shorter overall survival, and miR-216b-5p restoration reduces chemoresistance as evident by decreased cellular migration and promoted apoptosis [32]. You Y *et al*. have checked miR-216b-5p expression at a low level in pancreatic cancer, and further verified the tumor-suppressive activities of up-regulated miR-216b-5p for cell proliferation and anti-apoptosis phenotypes [33]. Mohammad-Nazir Menbari *et al*. make a great contribution to breast cancer management as to the suppressive role of miR-216b-5p for cellular proliferation and progression [12]. Conforming to these studies, we studied that miR-216b-5p expression dropped in NSCLC, and radiation-resistant NSCLC cells had impaired growth if overexpressing miR-216b-5p. miR-216b-5p poor expression has once been confirmed in hepatocellular carcinoma; TUG1/miR-216b-5p conforms to the mode of miR-sponge, and TUG1-mediated spongy effect on miR-216b-5p aggrandizes tumors to progress [34]. An academic record has implicated that in the context of prostate cancer, miR-216b-5p overexpression improves chemosensitivity while miR-216b-5p silencing enhances chemoresistance in Linc00518-naïve cells [15]. miR-216b-5p is down-regulated in cervical cancer, and miR-216b-5p inhibition is capable to offset cell proliferation blockade induced by LINC00152 deficiency [14]. As for colorectal cancer, an experimental observation displays that the oncogenic activities of LINC00265 could be counter-acted by ectopic miR-216b-5p [35]. Showing consistency with the reports, our analysis offered evidence that LINC01578 could sequester miR-216b-5p, and miR-216b-5p inhibition in turn obstructed LINC01578 depletion-induced prevention of cancer development.

TBL1XR1 is one of the chronic obstructive pulmonary disease-related genes [36]. A current publication has announced the increase of TBL1XR1 expression in NSCLC, and evidenced that TBL1XR1 benefits cell survival and proliferation [17]. Additionally, it is known that TBL1XR1 knockdown refers to a strong suppressor for proliferation and anti-apoptosis activities of pancreatic ductal adenocarcinoma cells [37]. Our investigation also conformed TBL1XR1 up-regulation in NSCLC and determined the inhibitory impact of silenced TBL1XR1 on radiation-resistant NSCLC cell growth. miRNA/mRNA sequencing involves in lung squamous cell carcinoma development [38] . Based on that, we validated the targeting relation between miR-216b-5p and TBL1XR1, and ensured the fact that in radiation-resistant NSCLC cells modified by depleted LINC01578, TBL1XR1 overexpression stimulated cell development.

In our study, predicted by the bioinformatics website (https://starbase.sysu.edu.cn/agoClipRNA.php), there was a binding site between LINC01578 and miR-216b-5p, and miR-216b-5p had a targeting relationship with TBL1XR1. Meanwhile, the existence of the targeting relationship was also confirmed by the dual luciferase reporter gene assay and RIP assay. To detect the correlation between lncRNA LINC01578, miR-216b-5p and TBL1XR1, we performed Pearson analysis, and the results showed that LINC01578 was negatively correlated with miR-216b-5p, miR-216b-5p was negatively correlated with TBL1XR1, and LINC01578 was positively correlated with TBL1XR1. The mechanism of up-regulation of lncRNA LINC01578 in NSCLC cells may be due to the binding of histones, transcription factors to the promoter region of LINC01578, enhancing its activity, and further activating the expression of LINC01578 [39].

## Conclusion

In summary, our investigation results present that LINC01578 s an inducer of cell growth under radiation treatment, and increases radiation resistance. The mechanism concerning to LINC01578 in radiation resistance in NSCLC is related to miR-216b-5p/TBL1XR1 sequencing, forming a novel lncRNA-miRNA-mRNA interplay in cancer progression. In the present work, we did not find a strong correlation between LINC01578, miR-216b-5p and TBL1XR1 in the TCGA data, which requires further analysis. Also, signal pathways mediated by the new axis were insufficiently explored and we will further investigate it in the upcoming years.

## Supplementary Material

Supplemental Material
