# Supplementary material for: LINC01578 affects the radiation resistance of lung cancer cells through regulating microRNA-216b-5p/TBL1XR1 axis
Source: Bioengineered. 2022 Apr 27;13(4):10721–33. doi: 10.1080/21655979.2022.2051881 (PMC9208508; doi:10.1080/21655979.2022.2051881)
Supplement: Supplemental Material [file KBIE_A_2051881_SM3463.docx]

**Supplementary Table 1** Primer sequences used in quantitative PCR

| Gene | Sequences |
| --- | --- |
| LINC01578 | F: TGCGTATGACCTCTTGAAATGT |
|  | R: TCGGCAACTACAAAGGCCAA |
| miR-216b-5p | F: AAATCTCTGCAGGCAAATGTGA |
|  | R: Universal primer |
| TBL1XR1 | F: CTAGCACCTTAGGGCAGCATAAAG |
|  | R: GTCTTGTCTACTCCAGCACTTAGG |
| U6 | F: ATTGGAACGATACAGAGAAGATT |
|  | R: GGAACGCTTCACGAATTTG |
| GAPDH | F: ATGCTGCCCTTACCCCGG |
|  | R: TTACTCCTTGGAGGCCATGTAGG |

Note: F, forward; R, reverse; miR-216b-5p, microRNA-216b-5p; TBL1XR1, Transducin (beta)-like 1 X-linked receptor 1; GAPDH, glyceraldehyde-3-phosphate dehydrogenase.

**Supplementary Table 2** Effects of LINC01578 on clinicopathological parameters of NSCLC patients

| Parameter | Case | LINC01578 | | *P* value |
| --- | --- | --- | --- | --- |
|  |  | High expression  (n = 60) | Low expression  (n = 60) |  |
| Age (years) | | | | 0.355 |
| < 60 | 50 | 22 | 28 |  |
| ≥ 60 | 70 | 38 | 32 |  |
| Gender | | | | 0.270 |
| Male | 67 | 30 | 37 |  |
| Female | 53 | 30 | 23 |  |
| Tumour size (cm) | | | | 0.028 |
| > 5 | 63 | 38 | 25 |  |
| ≤ 5 | 57 | 22 | 35 |  |
| Lymph nodes metastasis | | | | 0.017 |
| YES | 54 | 34 | 20 |  |
| NO | 66 | 26 | 40 |  |
| TNM stage | | | | 0.028 |
| I-II | 65 | 26 | 39 |  |
| III-IV | 55 | 34 | 21 |  |

Note: Data were analyzed by chi-square test.
